# Refractory otitis media as the first manifestation of Wegener's granulomatosis

**DOI:** 10.1590/S1808-86942010000400024

**Published:** 2015-10-19

**Authors:** Bruno Almeida Antunes Rossini, Eduardo Amaro Bogaz, Fernando Kaoru Yonamine, José Ricardo Gurgel Testa, Norma de Oliveira Penido

**Affiliations:** a Graduate student - Department of Otorhinolaryngology of the Federal University of São Paulo (EPM-UNIFESP); b Graduate student - Department of Otorhinolaryngology - EPM-UNIFESP; c Graduate student - Department of Otorhinolaryngology - EPM-UNIFESP; d Professor - Department of Otorhinolaryngology - EPM-UNIFESP; e Professor - Department of Otorhinolaryngology - EPM-UNIFESP

**Keywords:** wegener's granulomatosis, antibodies, suppurative, otitis media

## INTRODUCTION

Wegener's Granulomatosis (WG) is an idiopathic systemic granulomatous vasculitis, probably of autoimmune origin, characterized by necrotizing granulomas in the lower airways, genitourinary tract and upper airways (UAW), associated with some degree of disseminated vasculitis.

Having an incidence of up to 3/100,000 inhabitants, its evolution can be indolent or fulminant and, if left untreated, it has a mortality rate of 82% in one year.

The UAW involvement happens in about 90% of the cases at some stage of the disease, and the oral cavity is the most commonly affected site. We hardly ever find suppurative otitis media as initial manifestation^1^, ^2^, ^3^, ^4^.

## CASE REPORT

A 42-year-old Caucasian woman, with intense otalgia and purulent otorrhea on the right side for 45 days, had a disorder which was refractory to the use of numerous antibiotic agents (amoxicillin-clavulanic acid, ciprofloxacin and ceftriaxone), associated with systemic and topical steroids. She developed vertigo, headache and mixed-severe right side hearing loss.

As prior history, she had renal dysfunction of unknown origin and a kidney transplanted sister.

She had central perforation on her right-side tympanic membrane, where we could see a red tumor suggesting a polyp and purulent otorrhea. The other otorhinolaryngological exams were normal.

Laboratorial findings showed an otorrhea secretion with the growth of pneumococcus sensitive to the antibiotic agents used, CBC showing neutrophilia and left side shifting, increased VHS and proteinuria.

**Figure 1:**
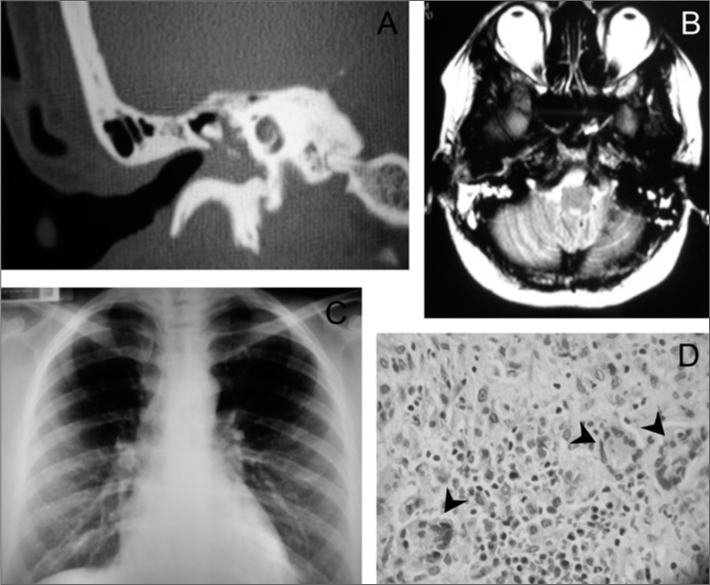
A- Right-side temporal bone CT-scan, showing a blurring of the tympanic cavity; B-Skull MRI in T2 with hyperintensity signal on the right-side mastoid; C- Chest X-Ray with reticulonodular infiltrate in the bases of the lung; D- histopathology study showing the granulomas (arrows).

Temporal bone CT-scan (Figure 1A) showed blurring of the tympanic cavity and of the mastoid cells; and skull MRI (Figure 1B) showed a hyperintensity signal in T2 on the right mastoid bone.

Since there was no clinical response, tympanomastoidectomy was indicated, together with the placement of a ventilation tube on the right side with the goal of eliminating the inflammatory process and to collect material for histopathology.

On the seventh postoperative day, the patient developed progressive ipsilateral peripheral facial paralysis, and she also had a worsening on her left-side hearing, stress dyspnea and mucous and bloody rhinorrhea. Nasal-fibroscopy also showed ulcerations covered by fibrin in the bilateral torus tubarius and on the lateral wall of the right nasal cavity.

Chest x-ray (Figure 1C) showed a reticulonodular infiltrate in the bases of the lung.

Histopathology showed the presence of granulomas (Figure 1D). We then ordered the anti-neutrophil cytoplasmic antibody (ANCA-c), which was positive all the way to 1/160 titer, confirming the diagnosis of WG.

Then we started treatment with deflazacort and cyclophosphamide, and the patient had an important improvement in her general health and a progressive recovery of her hearing and facial paralysis.

Since then, for about 21 months, the patient has been receiving treatment with cyclophosphamide with disease remission.

## DISCUSSION

In order to properly diagnose WG, one must have a high degree of suspicion, especially in patients with refractory disease, with the involvement of numerous organs and systems and worsening in the general status.

Besides detailing the clinical situation, one must order a chest x-ray and biochemical tests, including serology tests.

Biopsy with histopathology is the gold standard. In the head and neck region, often times it is necessary to obtain a proper sample. This should show unspecific inflammatory granulomas, with gigantic cells, small-vessel vasculitis, irregular necrosis areas and the presence of an acute and chronic inflammatory process coexisting.

ANCA-c has a sensitivity of 90% and a specificity of 99%, and the disease activity is associated the higher titers.

Treatment requires a multidisciplinary approach and is based on the use of immunosuppressive medication such as cyclophosphamide, methotrexate and high doses of steroids.

Associated with that, there are clinical and surgical measures which aim at reducing morbidity, improving the life quality of those with the disorder^3^, ^4^, ^5^, ^6^.

## FINAL REMARKS

Because of the high involvement of UAWs in WGs and sometimes manifesting early and atypical manifestations, such as in the case hereby described, the ENT physician plays a determining role in establishing a correct diagnosis and immediate treatment, thus crucially working towards a diagnostic change for those with the disease.
